# Editorial: Innovative Therapies in Bone Biology: What Can Be Learned From Rare Bone Diseases?

**DOI:** 10.3389/fendo.2022.928667

**Published:** 2022-06-09

**Authors:** Elisabeth M. W. Eekhoff, Teun J. de Vries, Ralph J. B. Sakkers, Wim Van Hul

**Affiliations:** ^1^ Department of Internal Medicine, Section Endocrinology, Amsterdam University Medical Center Utrecht (Amsterdam UMC), Amsterdam Bone Center, Vrije Universiteit Amsterdam, Amsterdam, Netherlands; ^2^ Department of Periodontology, Academic Centre for Dentistry Amsterdam, University of Amsterdam and Vrije Universiteit, Amsterdam, Netherlands; ^3^ Department of Orthopedics, University Medical Center (UMC) Utrecht, Utrecht, Netherlands; ^4^ Center of Medical Genetics, Antwerp University Hospital, University of Antwerp, Antwerp, Belgium

**Keywords:** rare bone disease, innovative, innovative, therapies, monitoring, collaboration

## Introduction

Due to their rarity and heterogeneity, rare bone diseases have been overlooked in recent decades, but the tide is turning. New developments in research models [see also the related, simultaneously hosted Research Topic “Innovative Models in Bone Biology: What can be Learned from Rare Bone Diseases?” (1)] and genetics help to gain more insight into the pathogenesis. But there is still a long way to go before effective treatments will be found for the various rare bone diseases.

In this Research Topic, 13 articles describe innovative and up-to-date options to improve the present treatment of rare bone diseases, including organization of care, setting up partnerships, innovative treatment monitoring, reuse of existing therapies and the developmental route to new therapies for various rare bone diseases.

## How to Organize Patient Care and Enter Into Partnerships to Enable Optimal Care and Support for New Treatment Options?

Due to the often extreme manifestation of rare bone diseases, collaboration of dedicated specialists and researchers with specialized expertise targeting this small number of patients is necessary to improve good clinical care. An organizational form that can achieve such shared goals through a committed, valued mentality of the employees is the organizational collaboration model, a good working example of which is described in a stimulating way in the article “Collaboration on rare bone diseases leads to the Unique Organizational Drive of the Amsterdam Bone Center’’ (Eekhoff et al.).

## Innovative Monitoring Options of New Treatments

Change in bone properties during a new therapy can be monitored *in vitro* by analyzing sequential bone biopsies. (Treurniet et al.) provide an update on new options to characterize and explore these biopsy features. A summary of the current numerous techniques is elegantly described. Interestingly, it is now also possible to directly evaluate some properties of bone that contribute to bone strength, *in vivo*. This is possible with impact micro-indentation (IMI). Schoeb et al. describe its current developments and applicability.

Unambiguous international methods and cut-off points/definitions for recording clinical measurements is of great importance. This becomes clear in an interesting overview of the current knowledge and clinical implications of hyperkyphosis on health by Koele et al. They show that when not using a fixed standard definition, the power of pooling many research results is complicated and thus hinders interpretation of the various studies. When not using the same standard definition, it is hard to compare (intervention) studies on hyperkyphosis.

## New Treatment Options for Rare Bone Diseases by Reuse or by Innovative Approaches

Following a previously described concept, in a bone graft-implant study, commonly used medication was successfully and innovatively re-applied by Bhadada et al. for pain relief in a patient with fibrous dysplasia. In this study, intralesional administration of bisphosphonates was found to be more effective than intravenous administration. Likewise, bisphosphonates had a positive treatment effect in patients with sternocostoclavicular hyperostosis. Here, intravenous pamidronate was effective in reducing pain and improving shoulder function and also led to decreased bone turnover on skeletal scintigraphy Leerling et al. 

While great progress can be made in understanding the pathogenesis of a disease, developing effective therapy can still be lagging behind, which has been elegantly described by Ralston and Gaston. They wrote a comprehensive review of current knowledge and future treatment options for Osteogenesis Imperfecta (OI). While explaining the shortcomings that remain in the current treatment, possible new treatment options were put into perspective, including the ongoing “reuse” studies and the status of new drug development.

Kloen et al. describe a combined surgical approach to address a non-union fracture along with a novel patellar fracture in a patient with OI. These fractures are among the rare injuries associated with a disrupted quadriceps extension mechanism in patients with OI. They put the result of this operation in the context of a literature review, which encourages to consider new combined approaches in OI surgery, if needed.

By monitoring treatments given for other concomitant diseases such as cancer, there is also a lot to learn about its effect on the underlying rare bone disease itself, as is the case with radiotherapy in fibrodysplasia ossificans progressiva (FOP). In this way, based on a case study and literature, possible future radiotherapy options for FOP lesions have been put into perspective by Botman et al.

In addition, Botman et al. showed in a second article that in emergency situations, surgery can be mandatory for an FOP patient. Such decisions require stringent innovative collaboration between various experts.

## New Challenging Solutions for Rare Bone Diseases

An example of a new bold procedure to solve a clinical problem in a patient with Gorham-Stout disease is presented in the article by de Keyser et al. In this article the authors give an interesting review on the current knowledge of this very rare bone disease for which still no real or proven treatment options exist.

## Innovative Research and Collaborative Approaches to Find a Cure for Rare Bone Diseases:

At present, the importance of different types of generated mouse models, human cell models and mutual collaboration in research is of great importance to find a good and effective therapy for various complex rare bone diseases. This also concerns fibrous dysplasia and McCune Albright syndrome, which are caused by a postzygotic mutation in the *GNAS* gene, leading to mosaic expression. The background of this rare bone disease, the current state of research and the difficulty and importance of finding a better therapy is explained and summarized by Lung et al.

A 1-week international scientific workshop on FOP provided by and according to the concept of the Lorentz Center has led to a concise overview of current knowledge and scientific gaps, described by de Ruiter et al. Such workshops are of vital importance in rare bone disease research, since bringing all researchers on a rare bone disease together provides an effective platform to update each other on the latest research. It further contributes to a clear roadmap for future research, joint efforts and the scientific steps to be taken to find a cure for FOP. This workshop is an example of how international cooperation can be promoted.

## Conclusion

The 13 contributions to this Research Topic on innovative therapies in rare bone diseases have highlighted the progress and developments of potentially new treatment options, treatment monitoring, collaboration and improvement of care in rare bone diseases.

It is clear that much research remains to be done. Only through increased knowledge of the underlying cellular processes, we can initiate new research models. These will lead to new drugs or to reuse of existing drugs. Above all, through collaboration, this can lead to novel treatment options to the benefit of the patient. The current topic provides a comprehensive overview of the status of some important rare bone diseases, with special emphasis on novel treatments. These described novelties may inspire others and can serve as an example or guideline for new developments in research, collaboration and care pathways in other rare bone diseases.

## Author Contributions

EE designed, wrote, and submitted the editorial. TV, RS and WH contributed to the design, writing and editing of the editorial. All authors contributed to the article and approved the submitted version.

## Conflict of Interest

The authors declare that the research was conducted in the absence of any commercial or financial relationships that could be construed as a potential conflict of interest.

## Publisher’s Note

All claims expressed in this article are solely those of the authors and do not necessarily represent those of their affiliated organizations, or those of the publisher, the editors and the reviewers. Any product that may be evaluated in this article, or claim that may be made by its manufacturer, is not guaranteed or endorsed by the publisher.
